# Are attention-deficit/hyperactivity disorder symptoms associated with negative health outcomes in individuals with psychotic experiences? Findings from a cross-sectional study in Japan

**DOI:** 10.3389/fpsyt.2023.1133779

**Published:** 2023-05-03

**Authors:** Andrew Stickley, Aya Shirama, Tomiki Sumiyoshi

**Affiliations:** Department of Preventive Intervention for Psychiatric Disorders, National Institute of Mental Health, National Center of Neurology and Psychiatry, Kodaira, Tokyo, Japan

**Keywords:** ADHD, comorbidity, depression, psychosis, suicidal ideation

## Abstract

**Objective:**

Although research has indicated that the prevalence of attention-deficit/hyperactivity disorder (ADHD) may be elevated in individuals with psychotic disorders, as yet, there has been comparatively little research on this association and its effects among adults at the subclinical level. To address this deficit, the current study examined the association between psychotic experiences (PE) and ADHD symptoms in Japanese individuals and whether the presence of ADHD symptoms increases the risk for negative health outcomes in people with PE.

**Method:**

Data were analyzed from an online sample of 1,452 individuals (age 18–89; 51.5% female) collected in 2021. Information on PE was obtained with the PRIME Screen-Revised (PS-R), while the Adult ADHD Self-Report Scale (ASRS) Screener was used to measure ADHD symptoms. Information was also obtained on a number of health outcomes including anxiety and depressive symptoms and suicidal ideation. Logistic regression was used to assess associations.

**Results:**

In a fully adjusted analysis PE were associated with almost three times higher odds for ADHD symptoms (OR: 2.92, 95%CI: 1.19–7.17). In an analysis that was restricted to individuals with PE, ADHD symptoms were associated with significantly increased odds for depressive symptoms, lifetime suicidal ideation, perceived stress and severe sleep problems.

**Conclusion:**

ADHD symptoms are present in some individuals with PE and increase the odds for several detrimental health outcomes in this population. Identifying co-occurring PE and ADHD/ADHD symptoms may facilitate treatment options and help prevent negative health outcomes in individuals with these conditions.

## Introduction

Psychotic experiences (PE) consist of subclinical hallucinations and delusions that occur among individuals in the general population (1). An earlier review study that used data from 61 cohorts found that the median estimated prevalence of PE was 7.2% in the general population (2), while more recent research has indicated that the self-reported lifetime prevalence of at least one PE may be substantially higher (16.5%) in some instances (3). PE have been linked to a variety of detrimental outcomes in adults including worse physical (4) and mental health (5). Regarding the latter, a study that used data from 18 countries that were collected in the World Mental Health Survey found that PE were linked to mood and anxiety disorders, eating disorders and substance use disorders in adults (5). More recent research has further shown that the co-occurrence of PE and common mental disorders increases the odds for negative outcomes such as poor physical functioning and suicidal behavior (6).

PE have also been linked to impulse control disorders (5). In particular, there is a growing body of evidence that PE are associated with attention-deficit/hyperactivity disorder (ADHD), which is a condition characterized by a persistent pattern of inattention and/or hyperactivity-impulsivity that negatively affects functioning in different settings (7). A recent study that used data from US individuals aged 8–21 found that ADHD was elevated in psychosis spectrum (PS) symptom youth vs. non-PS youth (45% > 20%) (8). In addition, cross-sectional research from South Korea and longitudinal research from England has also, respectively, linked psychotic-like experiences in adolescents with deficits in attention (9), and the ADHD combined subtype at age 7 to PE at age 12 (10). The association has also been observed in adults. A study that used data from the Adult Psychiatric Morbidity Survey 2007 found that a higher level of ADHD symptoms was associated with PE (11). Further, in the above-mentioned World Mental Health Survey study ADHD was found to predict the subsequent onset of PE (but not vice versa) (5). Several mechanisms might underlie the association between PE and ADHD. For instance, there may be a biological component; recent research has reported a shared genetic liability between PE and ADHD (12), while other authors have highlighted that altered dopamine (DA) levels may play a role in the development of both PE and ADHD symptoms (13). Alternatively, it is possible that other factors such as substance use disorder might also link ADHD and PE as recently hypothesized for the ADHD-psychotic disorder association (14).

This study will examine the association between PE and higher ADHD symptoms (i.e., possible ADHD cases that are deemed subclinical in the absence of a diagnosis) in Japanese individuals aged 18 and above and whether ADHD symptoms increase the risk for negative health outcomes in persons with PE. This research is warranted for several reasons. First, until now, there has been comparatively little research on PE in Japanese adults. This is an important omission as a recent study found that PE were linked to severe social withdrawal (*hikikomori*) in the Japanese general population (15) raising the possibility that they are associated with other detrimental outcomes in this setting and thus requiring further research. Second, it is important to study the relationship between PE and ADHD in a non-western setting to further elucidate the association – especially as not all studies from the West have found that ADHD increases the risk for PE (16). Third, given that an earlier study from Japan found that ADHD symptoms were associated with an increased risk for suicide attempts in outpatients with psychosis (17), it can be speculated that ADHD symptoms might also be important for negative outcomes in individuals with PE. However, to the best of our knowledge this issue has not been examined.

A focus on the association between PE and ADHD symptoms in Japan may be particularly instructive. Previous research has suggested that the stigma surrounding mental illness may lead to delays in seeking treatment for mild mental disorders in Japan, as well as a longer duration of untreated psychosis (18, 19). This might also explain why some research has indicated that there may be a large number of adults with undiagnosed and untreated ADHD in Japan (20). Although an earlier study found that attenuated psychotic symptoms were not associated with help-seeking behavior in Japan (21), other research has suggested that PE may serve as a general marker for a variety of adverse outcomes including more severe psychopathology (22), while ADHD symptoms have been associated with worse physical and mental health outcomes in undiagnosed Japanese adults (20). Given this, the current study had two main aims: (i) to examine if PE are associated with ADHD symptoms in Japanese individuals; (ii) to explore whether ADHD symptoms are important for negative health outcomes in people with PE. As previous research has indicated that both ADHD and ADHD symptoms may be more common in those with PE and that ADHD symptoms are themselves linked to worse physical and mental health, it was hypothesized that PE would be significantly associated with ADHD symptoms and that ADHD symptoms would be linked to worse health outcomes in individuals with PE.

## Methods

### Study sample

Data came from an online survey of the Japanese general population undertaken in late February 2021. The survey was administered by a commercial survey company, Macromill. A questionnaire was initially sent to 8,628 respondents that were drawn from the company’s online commercial web panel. An additional 1728 respondents were subsequently surveyed to increase the size of the final sample. After both phases of surveying were completed the final sample comprised 1,452 respondents who were selected based on three main demographic criteria – age (aged 18 and above), sex (representative of the total Japanese population) and residency area (respondents should be drawn from each of Japan’s 47 prefectures). Permission for the survey was provided by the Ethics Committee at the National Center of Neurology and Psychiatry, Tokyo, Japan (approval number: A2020-088). Informed consent was provided by all participants.

### Measures

#### Psychotic experiences

PE were assessed with the PRIME Screen-Revised (PS-R). This 12-item self-report scale measures (positive) psychosis symptoms: hallucinations, delusional ideas, persecutory ideas, unusual thought content, perceptual abnormalities, suspiciousness, and grandiose ideas (23). Items are rated on a seven-point scale from 0 (definitely disagree) to 6 (definitely agree). We used the scoring method suggested by the scale’s developers to categorize positive cases using three main criteria – (i) the strength of item agreement (a rating of 5 or 6) in conjunction with, (ii) the duration of the symptom, or (iii) having a total PS-R score of 39 or above. The PS-R has been previously validated in Japan (23).

#### ADHD symptoms

The Adult ADHD Self-Report Scale (ASRS) Screener was used to assess ADHD symptoms (24, 25). This 6-item screening scale enquires about inattention and hyperactivity symptoms in the previous six months using a 5-point response option that runs from never (scored 0) to very often (scored 4). The total score ranges from 0 to 24 with higher scores signifying increased ADHD symptoms. The Screener has been validated in numerous studies as being able to discriminate potential ADHD cases from non-cases (26) as well as for use in community epidemiological surveys (25). Following the suggestion of the scale’s developers, in this study a score of 14 and above was used to categorize higher ADHD symptoms (25). Cronbach’s alpha for the scale was 0.87.

#### Covariates/health outcomes

Information was also obtained on several sociodemographic and other health variables. Besides sex (male, female), information was also obtained on age which was subsequently categorized into three groups: 18–34, 35–59 and ≥ 60, representing young, middle-aged and older adults, respectively. For education level, individuals were categorized into those with a higher education (two-year college, university, graduate school) and those who had received less than a higher education (junior high school/below, high school, vocational high school). For marital status respondents were categorized as married or not married. The economic situation of respondents was assessed with a question that inquired about household financial income. This was measured in millions of yen and divided into three categories, (i) < 4 million, (ii) 4 < 10 million, and (iii) ≥ 10 million (106.55 JPY = U.S. $1 at the time of the survey). Given the large portion of respondents that failed to answer this question (22.7%) and our desire to keep as many individuals in the analysis as possible, a fourth (iv) ‘missing’ category was also created.

Self-rated health was categorized as either good/very good, fair, or poor/very poor. In the health analysis poor self-rated health (comprising both poor and very poor self-rated health) was used as the outcome. The occurrence of depressive symptoms in the past two  weeks was assessed with the self-report Patient Health Questionnaire (PHQ-9) (27). This scale has nine items that produce a total score between 0 and 27 with higher scores indicating greater depressive symptomatology. A cut-off score of 10 and above was used to categorize at least moderate depressive symptoms. The self-report Generalized Anxiety Disorder-7 (GAD-7) scale was used to assess anxiety symptoms in the past two  weeks (28). This 7-item scale produces a total score that can range between 0 and 21 with higher scores indicating increased anxiety symptoms. Following the suggestion of the scale’s developers a score of 10 and above was used in the current study to categorize at least a moderate level of anxiety (28). Perceived stress was assessed with the Perceived Stress Scale (PSS-14) (29). This self-report scale consists of 14 items that when summed produce a score that ranges from 0 to 56 with higher scores indicating greater stress. The scale has been validated in Japan previously (30). In the current study, the scale was used as a continuous score when employed as a covariate, while in order to focus on those individuals experiencing the highest levels of stress, the top decile of scores was chosen as a cut-off point (a score of 37 and above) when examining stress as a health outcome. Lifetime suicidal ideation was assessed with a question enquiring about suicidal thoughts that has been used in previous research (31). Severe sleep problems were assessed with a question which asked, “Overall in the last 30 days, how much of a problem did you have with sleeping, such as falling asleep, waking up frequently during the night, or waking up too early in the morning?” with five response options ranging from ‘none’ to ‘extreme’. Those who responded that they had either severe or extreme sleep problems (options 4 and 5) were categorized as having severe sleep problems. Loneliness was assessed with the Three-Item Loneliness Scale (32). Scores from the summed items create a total score that ranges from 3–9 with higher scores indicating greater loneliness. In this study, the top 15% of scores (a score ≥ 7) were used to categorize loneliness. Cronbach’s alpha for the scale was 0.70. Finally, those who agreed with the statement, “I feel that what happens in my life is often determined by factors beyond my control” were categorized as having a low locus of control. This item has been used previously by researchers when assessing perceived control (33).

### Statistical analyses

Descriptive statistics were first calculated for the study sample. Next, we examined whether the detrimental health outcomes were elevated in individuals with PE and those with ADHD symptoms compared to their counterparts without these symptoms with Chi-square tests used to assess differences. Logistic regression was then used to assess the association between PE and ADHD symptoms. Five models were used in the analysis: Model 1 examined the association between PE and ADHD symptoms. Model 2 included sociodemographic variables – sex, age, education, marital status and household income. Model 3 included the same variables as in Model 2 and also self-rated health. Model 4 included the same variables as in Model 3 and additionally included anxiety and depressive symptoms. The fully adjusted Model 5 included the same variables as in Model 4 and also included stress as a continuous score. Finally, restricting the analysis to those individuals who were categorized as having PE, we ran a series of separate logistic regression analyses to examine if ADHD symptoms were linked to seven detrimental health outcomes – depressive symptoms, lifetime suicidal ideation, perceived stress, severe sleep problems, poor self-rated health, loneliness and low locus of control. Given the low number of cases included in these analyses they were only adjusted for sex and age.

The analyses were performed with SPSS version 24. Results are presented as odds ratios (OR) with 95% confidence intervals (CI). The level of statistical significance was *p* < 0.05 (two-tailed).

## Results

The study sample consisted of 1,452 individuals aged between 18 and 89 (mean age: 51.6 years, SD: 18.1 years) with 51.5% of the sample being female. Details of the main, sociodemographic and health variables are presented in Table 1. More individuals were, respectively, categorized as having PE than ADHD symptoms (4.2% [*N* = 61] vs. 3.5% [*N* = 51]) while 23.0% (14/61) of those individuals with PE also had ADHD symptoms. The prevalence of all of the detrimental health outcomes was significantly higher in individuals with PE and ADHD symptoms (Table 2).

**Table 1:** Characteristics of the study sample.

Main/sociodemographic variables	*N* (%)	Negative health outcomes	*N* (%)
Psychotic experiences		Anxiety	
No	1,391 (95.8)	No	1,295 (89.2)
Yes	61 (4.2)	Yes	157 (10.8)
ADHD symptoms		Depression	
No	1,401 (96.5)	No	1,205 (83.0)
Yes	51 (3.5)	Yes	247 (17.0)
Sex		Lifetime suicidal ideation	
Male	704 (48.5)	No	948 (72.4)
Female	748 (51.5)	Yes	361 (27.6)
Age		Stress (Mean (SD))	27.1 (8.0)
18–34	322 (22.2)		
35–59	572 (39.4)	Severe sleep problems	
≥ 60	558 (38.4)	No	1,350 (93.0)
		Yes	102 (7.0)
Education			
Higher education	937 (64.5)	Self-rated health	
<Higher education	515 (35.5)	Good/very good	698 (48.5)
		Fair	586 (40.7)
Marital status		Poor/very poor	155 (10.8)
Married	890 (61.3)		
Not married	562 (38.7)	Loneliness	
		No	1,234 (85.0)
Household income (yen)		Yes	218 (15.0)
< 4 million	415 (28.6)		
4 million to <10 million	599 (41.3)	Low locus of control	
≥ 10 million	109 (7.5)	No	1,089 (84.5)
Missing data	329 (22.7)	Yes	200 (15.5)

ADHD, Attention-deficit/hyperactivity disorder; SD, Standard deviation.

**Table 2:** Negative health outcomes in Japanese individuals with PE and ADHD symptoms.

	No-PE	PE	*p*-value	No-ADHD	ADHD	*p*-value
	*N* (%)	*N* (%)		*N* (%)	*N* (%)	
Anxiety symptoms			< 0.001			< 0.001
No	1,261 (90.7)	34 (55.7)		1,278 (91.2)	17 (33.3)	
Yes	130 (9.3)	27 (44.3)		123 (8.8)	34 (66.7)	
Depressive symptoms			< 0.001			< 0.001
No	1,174 (84.4)	31 (50.8)		1,196 (85.4)	9 (17.6)	
Yes	217 (15.6)	30 (49.2)		205 (14.6)	42 (82.4)	
Lifetime suicidal ideation			< 0.001			< 0.001
No	928 (74.0)	20 (36.4)		937 (74.3)	11 (22.9)	
Yes	326 (26.0)	35 (63.6)		324 (25.7)	37 (77.1)	
Stress			< 0.001			< 0.001
No	1,270 (91.3)	37 (60.7)		1,287 (91.9)	20 (39.2)	
Yes	121 (8.7)	24 (39.3)		114 (8.1)	31 (60.8)	
Severe sleep problems			0.001			< 0.001
No	1,300 (93.5)	50 (82.0)		1,310 (93.5)	40 (78.4)	
Yes	91 (6.5)	11 (18.0)		91 (6.5)	11 (21.6)	
Poor self-rated health			< 0.001			< 0.001
No	1,240 (89.9)	44 (76.6)		1,249 (89.8)	35 (72.9)	
Yes	140 (10.1)	15 (25.4)		142 (10.2)	13 (27.1)	
Low locus of control			< 0.001			0.001
No	1,062 (86.1)	27 (48.2)		1,057 (85.2)	32 (66.7)	
Yes	171 (13.9)	29 (51.8)		184 (14.8)	16 (33.3)	

PE: Psychotic experiences; ADHD: Attention-deficit/hyperactivity disorder.

In Model 1 PE were associated with over nine times higher odds for ADHD symptoms (Table 3). Adjusting the analysis for sociodemographic variables and self-rated health reduced the OR and there was an especially large reduction in the OR (over 40%) when the mental health variables were included in the analysis. The OR was further reduced when self-perceived stress was included. Nonetheless, in the fully adjusted Model 5, PE continued to be associated with almost three times higher odds for ADHD symptoms (OR: 2.92, 95%CI: 1.19–7.17). Other variables that were positively associated with ADHD symptoms were depressive and anxiety symptoms and stress symptoms, while adults aged 35–59 had significantly reduced odds for ADHD symptoms compared with those in the youngest age group.

**Table 3:** Association between PE and ADHD symptoms in individuals in the Japanese general population (*N* = 1,439).

	Model 1	Model 2	Model 3	Model 4	Model 5
	OR (95%CI)	OR (95%CI)	OR (95%CI)	OR (95%CI)	OR (95%CI)
Psychotic experiences	9.53 (4.66–19.49)^***^	7.69 (3.54–16.69)^***^	7.20 (3.25–15.96)^***^	3.72 (1.56–8.88)^**^	2.92 (1.19–7.17)^*^
Sex					
Female		1.19 (0.64–2.19)	1.23 (0.66–2.29)	1.31 (0.67–2.55)	1.16 (0.59–2.30)
Age					
18–34		Ref.	Ref.	Ref.	Ref.
35–59		0.26 (0.12–0.54)^***^	0.22 (0.10–0.47)^***^	0.34 (0.15–0.77)^*^	0.30 (0.13–0.69)^**^
≥ 60		0.18 (0.07–0.45)^***^	0.16 (0.06–0.40)^***^	0.40 (0.14–1.14)	0.44 (0.15–1.26)
Education					
Less than higher education		0.87 (0.44–1.72)	0.77 (0.39–1.55)	0.83 (0.40–1.72)	0.81 (0.38–1.73)
Marital status					
Not married		1.45 (0.71–2.96)	1.22 (0.59–2.52)	1.11 (0.51–2.41)	1.07 (0.48–2.37)
Household income (Yen)					
≥ 10 million		Ref.	Ref.	Ref.	Ref.
< 4 million		0.67 (0.19–2.29)	0.59 (0.17–2.04)	0.56 (0.15–2.09)	0.48 (0.12–1.84)
4 < 10 million		0.50 (0.15–1.64)	0.48 (0.15–1.56)	0.63 (0.18–2.23)	0.52 (0.14–1.91)
Missing data		0.72 (0.21–2.45)	0.67 (0.20–2.26)	0.79 (0.21–2.93)	0.65 (0.17–2.49)
Self-rated health					
Good/very good			Ref.	Ref.	Ref.
Fair			1.88 (0.92–3.86)	1.66 (0.77–3.60)	1.36 (0.61–3.02)
Poor/very poor			4.11 (1.77–9.51)^**^	1.78 (0.72–4.42)	1.37 (0.53–3.52)
Depressive symptoms				7.46 (3.02–18.43)^***^	4.86 (1.91–12.37)^**^
Anxiety symptoms				4.06 (1.86–8.88)^***^	2.78 (1.23–6.26)^*^
Stress symptoms					1.09 (1.04–1.14)^***^

PE, Psychotic experiences; ADHD, Attention-deficit/hyperactivity disorder.

OR, Odds ratio; CI, Confidence interval; Ref, Reference category.

^***^p < 0.001, ^**^p < 0.01, ^*^p < 0.05.

When the analysis was restricted to individuals with PE, ADHD symptoms were associated with significantly higher ORs for four of the seven health outcomes examined – depressive symptoms (OR: 5.36), lifetime suicidal ideation (OR: 11.54), self-perceived stress (OR: 17.52), and severe sleep problems (OR: 7.34), while the association was of borderline statistical significance for low locus of control (OR: 4.36, *p* = 0.054) (Table 4).

**Table 4:** Association between ADHD symptoms and negative health outcomes in individuals with PE (*N* = 61).

	Depressive symptoms	Lifetime suicidal ideation	Stress symptoms	Severe sleep problems	Poor self-rated health	Loneliness	Low locus of control
	OR (95% CI)	OR (95% CI)	OR (95% CI)	OR (95% CI)	OR (95% CI)	OR (95% CI)	OR (95% CI)
Variable							
ADHD symptoms	5.36 (1.27–22.66)^*^	11.54 (1.23–107.94)^*^	17.52 (3.25–94.50)^**^	7.34 (1.60–33.59)^*^	1.73 (0.39–7.65)	1.93 (0.53–6.97)	4.36 (0.98–19.44)
Sex							
Female	1.79 (0.59–5.42)	3.75 (0.97–14.53)	2.17 (0.60–7.87)	2.67 (0.57–12.58)	4.48 (1.08–18.50)^*^	0.41 (0.13–1.27)	0.89 (0.29–2.75)
Age							
18–34	Ref.	Ref.	Ref.	Ref.	Ref.	Ref.	Ref.
35–59	0.57 (0.16–1.99)	0.28 (0.06–1.29)	0.58 (0.15–2.36)	1.69 (0.31–9.16)	1.55 (0.36–6.72)	0.74 (0.22–2.47)	1.76 (0.48–6.44)
≥ 60	0.67 (0.15–3.08)	1.08 (0.17–6.78)	0.50 (0.08–3.02)	2.02 (0.22–18.61)	1.47 (0.24–9.17)	0.25 (0.04–1.52)	2.43 (0.52–11.23)

PE: Psychotic experiences; ADHD: Attention-deficit/hyperactivity disorder.

OR: Odds ratio; CI: Confidence interval; Ref: Reference category.

^*^p < 0.05; ^**^ *p* < 0.01.

Finally, several sensitivity analyses were undertaken for the seven health outcomes. First, all of the regression analyses were also adjusted for location. Second, they were then adjusted for all of the sociodemographic variables, i.e., age, sex, education, marital status and household income. Third, they were subsequently adjusted for all of the sociodemographic variables and mental health (anxiety symptoms when depressive symptoms was the outcome and depressive symptoms in all other analyses). There were no changes in the results when controlling for location. When adjusting for all of the sociodemographic variables there was just one change – ADHD symptoms were now significantly associated with a low locus of control (OR: 6.67, 95%CI: 1.20–36.99). When adjusting for all sociodemographic variables and mental health variables ADHD symptoms continued to be significantly associated with perceived stress and severe sleep problems, while low locus of control was of borderline statistical significance (*p* = 0.064). In addition, the health analyses were also undertaken using the ADHD variable as a continuous score. This produced similar results to those obtained previously. Specifically, when adjusting for sex and age ADHD symptoms were significantly associated with depressive symptoms, stress, severe sleep problems and low locus of control, while the association with suicidal ideation was of borderline significance (*p* = 0.05) (data not tabulated).

## Discussion

This study used data from 1,452 individuals aged 18 and above collected from an online sample to examine the association between PE and ADHD symptoms in the Japanese general population. Results showed that there was a strong association between PE and ADHD symptoms. Specifically, in a fully adjusted logistic regression analysis individuals with PE had almost three times higher odds for ADHD symptoms. There was also evidence that ADHD symptoms may increase the risk for negative health outcomes in people with PE as when the analysis was restricted to those with PE, ADHD symptoms were linked to depressive symptoms, suicidal ideation, high levels of stress and severe sleep problems.

Previous research has shown that there is a high level of comorbidity between PE and mental health disorders (5, 34, 35) although until now, there has been comparatively little research that has focused specifically on the association between PE and ADHD and prevalence estimates have varied between the studies that have been undertaken. For example, in a study of 172 adolescents aged 11–12 years old with a lifetime history of PE 4.7% had ADHD (36), while among 262 children with an average age of 12.4 years, 22 had PE and 14% (*N* = 3) of these also had ADHD (37). In contrast, in a sample of over 6,800 individuals aged 8–21, among the 1,320 people with PS the prevalence of ADHD was 45% (8). Further, among 2,385 adults with lifetime PE collected during the World Mental Health Survey 5.1% had ADHD (5). Similarly, in a sample of 509 adults with ADHD with a mean age of 25.12 years 5.1% had screened positive for psychotic symptoms during a 10-year follow-up period (16). In the current study 23.0% of individuals with PE had ADHD symptoms. Comparing this figure with those from previous studies among adults is complicated by the fact that those studies examined PE in adults meeting DSM-IV criteria for ADHD. Nonetheless, it can be speculated that the prevalence of ADHD may be comparatively high in the current study because a screening instrument was used to collect information on this condition.

In a fully adjusted logistic regression analysis PE were associated with almost three times higher odds for ADHD symptoms. Previous studies among adults have produced mixed findings on the association between PE and ADHD showing that there was either no association (16), that ADHD predicted the subsequent onset of PE but PE did not predict later ADHD (5), and that delusion-proneness was correlated with ADHD symptoms in a sample of 925 men in Sweden aged 18 to 35 (38). Interestingly, in an earlier longitudinal study Hennig and coauthors found that ADHD at age 7 was associated with PE at age 12 in statistical analyses that were unadjusted and then adjusted for demographic and maternal factors. However, when the analysis was further adjusted for other mental health diagnoses the association became non-significant indicating that certain psychiatric disorders may be important for the association (10). There is some indication that mental health might also play a role in the current study as when anxiety and depressive symptoms were included in the analysis the OR for the association between PE and ADHD symptoms reduced sharply, although it remained statistically significant.

In an analysis that was restricted to individuals with PE, ADHD symptoms were associated with significantly increased odds for several negative health outcomes. Earlier research has shown that adults with PE may be at increased risk for outcomes such as depression (5, 34), suicidal ideation (39), stress sensitivity (40) and sleep problems (41). However, until now, there has been little attempt to focus on the mechanisms linking PE and worse health outcomes even though a previous study showed that co-occurring depressive symptoms and PEs increased the risk for negative outcomes (35). This study focused on ADHD symptoms not only because PE and ADHD/ADHD symptoms can co-occur (5, 11) but also because ADHD symptoms have themselves been associated with the same negative health outcomes that PE have been linked to (42–45) as was further confirmed in the analysis in Table 2 in this study. Hence, this study was designed to determine if the presence of ADHD symptoms further increased the risk for these outcomes in individuals with PE.

Given the observed associations it is possible that various mechanisms might link ADHD symptoms with negative health outcomes in individuals with PE. For example, a recent study found that ADHD symptoms were associated with the presence of more (severe) psychotic symptoms in PS youth (8). This may be important as there is some evidence that a higher number of psychotic symptoms may greatly increase the odds for negative outcomes such as suicidal ideation (39). Alternatively, factors that are inherent in ADHD may have also played a role. In particular, a recent study has suggested that sleep problems might represent an intrinsic feature of adult ADHD (46), while impulsivity has been found to positively correlate with depression and stress across different age groups (47). Common underlying factors may also underpin the observed associations. Specifically, the altered transmission of DA may play a role in the emergence of both PE and ADHD symptoms (13), while other research has proposed that changes in DA neurotransmission might also be important in the pathophysiology of depression (48).

## Limitations

This study has several limitations that should be discussed. First, the data analyzed in this study came from individuals in the Japanese general population who were not randomly sampled but rather participated in an online survey. This may have increased the risk for selection bias (49) linked for example, to issues such as internet access. Second, given the potential sensitivity associated with issues such as mental health it is possible that non-responding may have been an issue, although a recent study from Australia found that participants may be more likely to respond to questions on sensitive topics in online surveys (50). Third, due to the relatively small number of individuals with PE we were limited in the number of variables we could safely adjust for in the analyses when examining the association between ADHD symptoms and health outcomes in individuals with PE, given the commonly applied rule that a minimum number of 10 events is needed per variable in logistic regression (51). Being able to control for a larger number of variables would have affected the observed results (as indicated in the sensitivity analyses when fewer associations were significant when adjusting for all demographic variables and mental health). In addition, it is possible that some of these analyses were underpowered to detect associations, while the confidence intervals were wide indicating imprecision in the estimates obtained. Fourth, we lacked information on whether respondents had diagnosed mental health conditions such as psychotic disorder or ADHD. This might have been important as there is some evidence, for example, that ADHD medication may be linked to the occurrence of reduced suicidal behavior (attempts) (52). Fifth, due to an absence of data we were not able to determine the effects of potentially important factors in the association between PE and ADHD symptoms such as illicit drug use. For example, cannabis use has been associated with the occurrence of both PE and ADHD symptoms (53, 54) and has also been linked to worse health outcomes such as depression and suicidal ideation (55, 56). Finally, as the data were cross-sectional we were not able to determine the directionality of the observed associations.

## Conclusion

This exploratory study showed that PE are linked to ADHD symptoms in the Japanese general population and that in individuals with PE, ADHD symptoms are associated with negative health outcomes. Screening for PE in individuals with ADHD/ADHD symptoms and ADHD symptoms in people with PE and first-episode psychosis may help with developing treatment options and facilitating better outcomes in both populations.

## Data availability statement

The raw data supporting the conclusions of this article will be made available by the authors, without undue reservation.

## Ethics statement

Permission for the survey was provided by the Ethics Committee at the National Center of Neurology and Psychiatry, Tokyo, Japan (approval number: A2020-088). The participants provided their written informed consent to participate in this study.

## Author contributions

ASt had the study idea, analyzed the data, and wrote the main text. ASh liaised with the data collection company, discussed the analysis, and commented on the main text for intellectual content. TS supervised the project and critically reviewed and revised the manuscript. All authors contributed to the article and approved the submitted version.

## Funding

This study was supported by AMED Grants (21he2202007, 22dk0307114), Intramural Research Grant (2-3) for Neurological and Psychiatric Disorders of the National Center of Neurology and Psychiatry, and the Japan Health Research Promotion Bureau Grants (2020-B-08, 2021-B-01) to TS.

## Conflict of interest

The authors declare that the research was conducted in the absence of any commercial or financial relationships that could be construed as a potential conflict of interest.

## Publisher’s note

All claims expressed in this article are solely those of the authors and do not necessarily represent those of their affiliated organizations, or those of the publisher, the editors and the reviewers. Any product that may be evaluated in this article, or claim that may be made by its manufacturer, is not guaranteed or endorsed by the publisher.
